# “We come as friends”: approaches to social accountability by health committees in Northern Malawi

**DOI:** 10.1186/s12913-019-4069-2

**Published:** 2019-05-02

**Authors:** Elsbet Lodenstein, Joyce M. Molenaar, Christine Ingemann, Kondwani Botha, Jenipher Jere Mkandawire, Loan Liem, Jacqueline E. W. Broerse, Marjolein Dieleman

**Affiliations:** 1 Athena Institute for Research on Innovation and Communication in Health and Life Sciences, VU University and KIT Gender, De Boelelaan 1085, 1081 HV Amsterdam, The Netherlands; 2 Athena Institute for Research on Innovation and Communication in Health and Life Sciences Communication (VU University), De Boelelaan 1085, 1081 HV Amsterdam, The Netherlands; 3 Foundation for Children’s Rights, Mzuzu, Malawi; 4 Simavi, Naritaweg 135, 1043 BS Amsterdam, The Netherlands; 5 Athena Institute for Research on Innovation and Communication in Health and Life Sciences, VU University and KIT Health, De Boelelaan 1085, 1081 HV Amsterdam, The Netherlands

**Keywords:** Social accountability, Governance, Health committees, Health worker performance, Quality of care, Maternal health

## Abstract

**Background:**

In Malawi, as in many low-and middle-income countries, health facility committees (HFCs) are involved in the governance of health services. Little is known about the approaches they use and the challenges they face. This study explores how HFCs monitor the quality of health services and how they demand accountability of health workers for their performance.

**Methods:**

Documentary analysis and key informant interviews (7) were complemented by interviews with purposefully selected HFC members (22) and health workers (40) regarding their experiences with HFCs. Data analysis was guided by a coding scheme informed by social accountability concepts complemented by inductive analysis to identify participants’ perceptions and meanings of processes of social accountability facilitated by HFCs.

**Results:**

The results suggest that HFCs address poor health worker performance (such as absenteeism, poor treatments and informal payments), and report severe misconduct to health authorities. The informal and constructive approach that most HFCs use is shaped by formal definitions and common expectations of the role of HFCs in service delivery as well as resource constraints. The primary function of social accountability through HFCs appears to be co-production: the management of social relations around the health facility and the promotion of a minimum level of access and quality of services.

**Conclusions:**

Policymakers and HFC support programs should take into account the broad task description of HFCs and integrate social accountability approaches in existing quality of care programs. The study also underscores the need to clarify accountability arrangements and linkages with upward accountability approaches in the system.

**Electronic supplementary material:**

The online version of this article (10.1186/s12913-019-4069-2) contains supplementary material, which is available to authorized users.

## Background

In many countries, health facility committees (HFCs) are a key component of strategies to improve community-health worker relations and to ensure the availability, quality and acceptability of health services. HFCs are defined as “any formally constituted structure with community representation that has an explicit link to a health facility and whose primary purpose is to enable community participation with the aims of improving health service provision and health outcomes” (McCoy et al., 2012, p. 451) [1]. HFCs are also referred to as health centre and clinic committees, village health committees, or health centre management committees. Although the mandates, roles and capacities of HFCs differ according to context, they are frequently expected to provide a community-health worker platform to ensure the bottom-up integration of community preferences in planning and decision-making in service delivery and oversee the implementation of plans, the availability and quality of services and the use of facility resources [1–4].

Although the role of HFCs in the governance of health services and the monitoring of health workers is receiving increased attention, little empirical evidence is available on how they perform this role in low-and middle-income countries [4]. Also, the existing evidence on the effectiveness of HFCs in influencing health provision is mixed. Previous studies have shown that HFCs, through increased participation and monitoring, can contribute to reducing health worker absenteeism and waiting times [5], and improved relations between the community, service users and health workers in Zimbabwe [6], Kenya [7] and Malawi [8]. Other studies, however, show that HFCs have limited impact on health worker performance and on how services are provided in Kenya [9] or Uganda [9, 10]. The gap between expectations of HFCs in governance and their roles in practice requires more research. This paper aims to add to the empirical evidence on the role of HFCs in the governance of health services by analysing the case of rural Malawi. It assumes that HFCs, through their formal intermediary position between the community and health workers, constitute an essential channel through which citizens can provide feedback to health workers and demand service improvements or explanations for actions and service delivery failures. This paper uses the concept of social accountability to explore the approaches HFCs use to monitor the quality of health services, the ways in which they demand accountability of health workers and the factors that influence HFC practices in rural settings. The paper provides a lens for reflection for health workers, managers and policymakers on the expectations regarding the role of HFCs in governance and accountability and for targeting improvement efforts in Malawi and elsewhere. It starts with a brief overview of HFC characteristics in Malawi, followed by a conceptual framework to assess HFCs role as social accountability interfaces.

### Health facility committees in Malawi

Primary health care in Malawi is offered by a network of around 977 health facilities, including health posts, health centres and hospitals [11]. These are public-governmental facilities (60%) as well as private not-for-profit facilities of the Christian Health Association of Malawi (CHAM) (27%) and other providers (13%) [12]. Health centres have a catchment area between 5000 and 20,000 people and most of them provide a basic package of services, comprising child care, vaccination, family planning, antenatal and normal delivery care, prevention and treatment of sexually transmitted infections. These services are provided free of user fees.

HFCs in Malawi are known as health centre advisory committees (HCACs) for the health centre level and hospital advisory committee (HAC) for hospitals. As per government policy, both health centres and hospitals are supposed to install such committees. This paper focuses on HCACs at health centre level of which, on paper, around 450 exist in Malawi [11]. According to official guidelines, HCACs consist of 10 elected community representatives who serve two-year periods [13]. The committee appoints a chairperson and is expected to hold meetings every 2 months, assisted by one of the facility staff members who act as a secretary. Local authorities, such as local government councillors or traditional chiefs are not eligible for committee positions. Any member of the health facility staff can be invited to the HCAC meetings as considered relevant [13, 14]. In the Malawi Health Sector Strategic Plan, HCACs are considered important actors in the local governance of health services and their central role is to “help communities to demand the quantity and quality of services that they expect by monitoring the performance of health centres” (Ministry of Health, 2011, p. 90). The training manual describes the duties and responsibilities of HCAC in four broad domains (1) bridging the communication gap between community and health staff, (2) inspection of facility conditions and drug stock, (3) formulating recommendations on facility equipment, and (4) complaint management [13]. The complaint management role of HCACs in Malawi is explicitly defined. HCACs are to receive and collect complaints from community and hospital management; investigate and assess the justification of complaints; forward complaints and results of investigations to facility management and district health management teams (DHMT) [13, 14]. The Malawian Ministry of Health is supported by international donors to implement community participation policies and strengthen the capacities of the HCACs. The majority of health facilities in the country has established HCACs that perform their functions with varying degrees of intensity and effect, depending, among others, on the level of donor support in the different regions.

### Health facility committees and social accountability

The governance role assigned to HCACs in Malawi by the Ministry of Health can be approached from an accountability perspective. Tasks such as monitoring, inspection and complaint management are essential dimensions of an accountability relation as defined by Bovens (2007). Bovens’ framework posits that accountability requires three steps to function: (1) information about standards, responsibilities and expectations for behaviour and actions as well as information about actual performance through monitoring; (2) dialogue involving questioning, debating and passing judgment on the issues; and (3) consequences referring to the imposing of positive and negative sanctions on actors [15]. It is primarily retrospective as explanations and justifications for decisions and actions are sought after they have been observed [16]. Such retrospective processes draw attention to what did not work and to find solutions or remedies for failures in service delivery [17]. The primary objective of this process is to ensure that organisations and individuals carry out their responsibilities as formally defined or socially expected [18]. Some scholars question whether accountability requires all these elements for a relation to work in practice. Only the stages of information and debate might be sufficient to qualify a relationship as an accountability relation [15, 19]. Also, accountability is not necessarily a formal obligation or agreement; it may also be more intangible or voluntary where an actor feels a moral obligation to explain and justify his conduct to some significant other [20]. Accountable behaviour is then encouraged without procedures and the threat or application of sanctions.

This study assesses a specific type of accountability: social accountability which refers to citizens, in this case HFCs, seeking accountability from providers with regard to the relevance, accessibility, quality and equity of the services they provide (or fail to provide) [19, 21]. The three steps provide a useful analytical framework to understand accountability actors, responsibilities and interactions. Its applicability to citizen groups and HFCs, and in particular the latter aspect of imposing sanctions, is however questioned. In most contexts, citizen groups, including community representatives in HFCs, do not have the capacity for formal sanctions [19]. In Malawi, for example, the HCAC guidelines suggest practical ways to manage complaints and to engage community representatives and health workers to discuss health centre functioning. In such discussions, HCACs can expect answers or explanations of health workers for service failures, but they do not have the means of enforcement, defined as measures to ensure compliance to decisions [22]. There is disagreement in the literature whether this lack of formal power matters. Some authors consider it the main weakness of HFCs and citizen action more generally, leading to an inability to influence service delivery substantially [23, 24]. Others argue that the first two steps (information and dialogue), constituting the ‘soft’ aspects of accountability, can be sufficient contributors to responsible behaviour [18].

Apart from the formal mandate, other HFC features shape HFCs’ possibilities to influence health workers’ practices, including skills and resources to perform their tasks [1, 4, 24, 25] and recognition and support from health providers, health workers and communities. Joshi (2014) asserts that the dynamics of social accountability are best understood within their micro-context [26]. Local actors’ understanding of their governance context and their sense of agency, likely shape their actions [27]. In many settings, for example, the promotion of quality of care is seen as a responsibility of health managers or government, and disrespect in maternal healthcare is regarded as a failure of malfunctioning health workers, generating a sceptic attitude towards the role of HFCs in the monitoring of health workers [28].

## Methods

### Study setting and site selection

This study is part of a larger research project on social accountability in maternal health service delivery in Mzimba North and South districts, situated in the Northern Region of Malawi. Data collection took place between April 2015 and June 2016. The district was purposefully selected in the context of a partnership between the researchers, an NGO and district authorities. Ethical approval was obtained from the National Health Science Research Committee of the Ministry of Health in Malawi (NHSRC#15/03/1398).

Our study goal was to gain a comprehensive understanding of experiences with, and perceptions on, the role of HCACs as social accountability interfaces and the approaches HCACs use to address poor service quality and performance in rural health centres. Although HCACs oversee all services offered by health centres, the focus of this study was on maternal health services. A total of 41 HCACs were eligible in Mzimba district, based on their association to a rural health centre (not urban health centres or rural health posts) and a minimum level of functionality (according to district health authorities). Out of these 41 HCACs, we selected all HCACs in the Northern part of the district (*n* = 12) and a sample of HCACs in the Southern part of the district. This district is divided into six clusters of health centres that are constructed by the DHMT for their supervision visits on the basis of the geographical concentration of health centres. Out of the six clusters, we randomly selected three that hosted a total of 10 HCACs associated with rural health centres, resulting in 22 included sites.

The proportion of births assisted by skilled health personnel in the Northern Region is reported to be 90.6% in 2015 [29]. Maternal healthcare, like most other care, is provided free of user fees. The health centres in the study sites had an average of two skilled birth attendants, below the planned four. The number of deliveries per year per health centre (2014) ranged from 36 to 723 with a mean of 326 deliveries per year.

### Data collection

The goal of data collection was to gather evidence from multiple sources on social accountability approaches, perceptions, outcomes and contexts associated with these approaches. In each HCAC site, we collected available minutes from HCAC meetings, letters pertaining communication between HCAC and health workers and we conducted semi-structured interviews with HCAC members, health workers and District Health Management Team (DHMT) members. A facility checklist was used to document the conditions in the health centre and evidence on the presence of social accountability tools, such as scorecards, monitoring sheets, complaint and suggestion boxes, or a phone number to call in case of a complaint. Two key informants, an NGO representative and a researcher, provided guidance and feedback on the data collection tools, choice of participants and interpretation of data during analysis. They were both community development professionals and had experience with the set-up and training of HCACs.

The interview guides for both HCAC and health workers covered a range of topics including a general section on perceived roles and functioning of HCACs and a specific section to obtain perceptions on, and examples of, approaches to the monitoring of quality of services, complaint and feedback processes. The guide consisted mostly of open questions but also included some statements with yes/no answers for probing purposes (e.g. ‘HCACs should monitor health workers’) and some closed questions to cover HCAC characteristics. Concepts from Bovens’ conceptual framework were combined with concepts as defined in the HCAC training manual, in particular on complaint and feedback management (see Table 1). Scans from relevant documents were obtained from HCAC chairpersons and secretaries. We collected a total of 12 HCAC meeting minutes. Additional interviews were held with DHMT members to get their views on, and experiences with, HCACs in relation to the performance of health workers. Questions included their role in supporting HCACs, and supervision and accountability relations in the district health system. The DHMT shared copies of minutes of three district-level meetings on HCAC, and two copies of letters from HCACs addressed to them.

**Table 1:** Interview guides

Specific sections HCAC	Specific sections health workers	Shared section HCAC and health worker guide
Composition and roles of the HCAC	Experiences with receiving feedback from community members and the HCAC specifically	Perceived advantages of the HCAC for service users (women) and health workers
Activities to monitor the quality of care	Preferred roles of the HCAC and perceptions on the role of HCACs as social accountability interfaces	Suggestions for points for improvement of the relation between HCACs, health workers and the community and the roles of HCAC as a social accountability interface
Complaint and feedback management by the HCAC		

The data collection at health centre level was divided between two researchers (JM and CI). During 2 months, they jointly visited all sites. The travel schedule to visit health centres was set based on the participants’ availability, taking into account the accessibility and relative distance between health centres. In each site, two or three interviews were held, one with the HCAC chairperson and one or two with health workers. Interviews were conducted with informed consent and lasted a maximum of 60 min. Interviews with most HCAC members were in the local language Tumbuka, assisted by a translator while interviews with health workers were in English. Interviews with DHMT members were divided between the three researchers (EL, JM, CI).

### Selection and recruitment of study participants

In each site, participants were purposefully sampled to represent the HCAC, health centre management (the officer in-charge who is usually a clinician and the contact person for the HCAC) and health workers (registered or enrolled nurse-midwives). Nurses were targeted because they are most involved in maternal healthcare, the focus of the larger research project. The DHMT provided contact data of the HCAC chairperson and the health centre in-charge and participants were invited by phone or letter. Table 2 provides an overview of the interview participants. A total of 62 interviews were conducted in 22 health centre sites.

**Table 2:** Participants

HCAC members	22 participants	Gender: Male: 19 (86%) Female: 3 (14%)	Role in HCAC: Chairperson: 15 (68%) Vice-chairperson: 4 (18%) Treasurer: 2 (9%) Member: 1 (5%)
Health workers	40 participants	Gender: Male: 22 (55%) Female: 18 (45%)	Profession: Clinician: 16 (40%) Nurse: 24 (60%)
DHMT members	7 participants	Gender: Male: 3 (43%) Female: 4 (57%)	Function: District Health Officer: 2 District Nursing/medical Officer: 2 Human Resource Officer: 1 HCAC officer: 1 CHAM deputy director: 1

### Data analysis

During data collection, observations on interview data and emerging findings per site were noted down. Regular reviews were conducted between the three researchers (JM, CI and EL) to check consistency in data collection. All interviews were digitally recorded after consent and transcribed by local transcribers and the researchers. Interviews in Tumbuka were translated into English. The field notes, transcripts and documents were all uploaded in the qualitative data analysis program Maxqda (version 11). We used deductive thematic analysis methods during the first phase of data analysis, guided by Bovens’ concepts of an accountability process, applied to all data sources. This analysis supported the identification and analysis of patterns in the ways in which HCACs facilitate social accountability and the outcomes associated with this process [30]. Three researchers did this separately for each available data source per HCAC and according to a codebook with predefined codes in the MaxQDA database. Data from all sites were then combined as the focus was on identifying common patterns and descriptions rather than differences. The three researchers discussed the common themes, and EL integrated the analysis to a final set of themes and sub-themes. EL also performed a second analysis on context, including the identification of perceptions, explanations and motivations of participants and contextual data in the interview and documentary data.

## Results

This section starts with a presentation of the main features and activities of HCACs in the study area, followed by a description of how participants generally conceptualised the role of HCACs. Participants’ accounts of how HCACs identify and address poor health worker performance are then presented in terms of three major themes generated by the analysis: monitoring performance, “sitting down” and mediation, and reporting. This is followed by an analysis of participants’ perceived value and challenges of HCACs as social accountability interfaces.

### Main features and role perceptions of the HCACs

#### Composition and training

An additional file provides an overview of the features of the 22 HCACs included in the study [see Additional file 1]. The majority of HCACs have 10 members representing the community, with often male chairpersons and an underrepresentation of women. Female members often have the role of treasurer, in some instances of vice-chairperson and once of chairperson. In half of the committees, individual members represent one area or village around the health centre; the other half is composed of a mix of representatives from the traditional leadership (chief), religious bodies, schools, youth groups, and community-based organisations. The health centre officer in-charge fulfils the role of HCAC-secretary in 50% of the HCACs, but in his/her absence another health worker stands in. The other 50% of the HCACs hold their meetings without the presence of a health worker or invite one only if needed. HCAC members do not receive a salary; some of them, however, irregularly receive allowances for participation in training sessions and health campaigns. The majority of the HCACs had received training within the past 2 years, provided by DHMT members in cooperation with NGOs. The most recent HCAC training includes modules on roles and responsibilities of the HCAC, drug management, leadership, conflict management, record keeping and action planning. Within the sample of 22 HCACs, five were inactive because they were just established or there was a lack of initiative from leaders and health staff to meet at the facility or to hold statutory meetings. Hence, most results are based on data from 17 active HCACs.

#### Statutory meetings

HCACs formally meet once a month on average, but the frequency of their meetings is influenced by climatic conditions, social events, public health events, training, emergencies or campaigns organised by the health centre, the Ministry of Health or NGOs. Therefore, the content of meetings may be a more meaningful indicator of their functioning than the frequency of their meetings. According to 12 meeting minutes obtained and respondent accounts, the development of the health centre constitutes a priority for all HCACs as it headed the list of topics that HCACs addressed in their most recent meeting. The development of the health centre involves the mobilization of resources (money, material) from community chiefs for the reparation and construction of health centre infrastructure such as health worker houses, maternity wards and guardian shelters. A few HCACs advocate for resources beyond the community level: they engage with politicians, local authorities and DHMT to mobilise human and financial resources for the health centre, fostering an enabling environment for the basic delivery of healthcare tasks. Nine HCACs had governance and performance related topics on their meeting agenda such as health worker absenteeism, long waiting times, lack of transparency in the reception of drugs, rude behaviour, poor communication and verbal abuse by patients and health workers, as well as frictions among health workers. This suggests that poor health worker performance is being discussed and documented, even in cases where the officer in-charge is the secretary.

#### General perceptions on the role of HCACs

Participants recurrently used two metaphors to describe the HCAC: “bridge” and “parent”. Bridge refers to the relation between the community at large and the health centre, while parent refers to the personal and individual relations between HCAC members and health workers.

“Bridge” appears to be a well-known and practised term that participants associated with health service promotion. Health service promotion in maternal healthcare included informing women, their families and community leaders about the importance of facility-based deliveries and the risk of home deliveries. It also included presenting the health centre as a pleasant place with friendly health workers who should not be feared. At a more personal level, both HCAC members and health workers considered the HCAC as a “parent”, “father” or “mother” of the health worker. Four HCAC members stated that they need to receive and treat health workers in the community as their children, as they are often temporarily posted far away from their home towns:

*“We are the parents of the health workers because for health workers here is not their home. They are employed by the government, they are here like visitors. We are the owners of the place, and we need to help in problem-solving for the visitors; the owners need to be close and be able to provide quick assistance so that the visitors stay comfortable”* (HCAC member).

HCAC members further associated “parental” support with encouraging and complimenting health workers on their good work and listening to their concerns, advising them on how to behave according to community customs and accompanying them to local events and funerals. Health workers interpreted the support role of HCACs in terms of providing protection from violent or demanding patients, defending health worker behaviour in front of patients, and intervening when patients quarrel around the health centre. Both HCAC members and health workers see HCACs as important supporters of the internal organisation of health centres by mediating conflicts among health workers and by advising staff to “work in unity”. It is against this background that social accountability efforts by HCACs should be understood.

### Approaches HCACs use to identify and address poor health worker performance

As suggested in the introduction, (social) accountability is practised in a cycle of monitoring performance, dialogue and consequences (sanctions). In the following paragraphs, we first analyse how, and to what extent, HCACs monitor service delivery. This is followed by the approaches they use to address poor performance in dialogue with health workers and through reporting to health authorities.

#### Monitoring performance

Half of the HCACs report being present at the health centre at least twice a week to support patients and health workers and to monitor the provision of services: *“see how patients are treated”* and *“check how health workers are working”.* Five HCACs make sure one of the members is present daily; two rotate visits among members to give all members *“a chance to come and see”.* Two HCACs use a written form to keep track of their observations, for example on health worker duty hours and consultation time; the other HCACs do not use such documentation tools. Four HCACs do not regularly visit the health centre for monitoring purposes. The chairpersons of two of these HCACs explained that this was due to a lack of training and information; they did not feel entitled to monitor health workers. All health workers agreed that HCACs should collect patients’ opinions and 75% agreed that HCACs should monitor the quality of maternal healthcare:

*“Because the HCAC chairman, it is their responsibility to see how people are helped at the health centre. Are people there being helped timely or has the health worker been punctual, because all those issues are in the strategies of the HCAC”* (male health worker and in-charge).

HCACs identify poor health worker performance through these visits and complaints received from patients and communities. None of the health centres had a suggestion box or names or contact details of HCAC members displayed, and none of the HCACs systematically approach patients to ask about the services they received. Rather than pro-actively soliciting patient feedback, women, their guardians or relatives, community health workers and chiefs approach HCAC members themselves to express their opinions and complaints, but also compliments, about the quality of services.

**Fig. 1:**
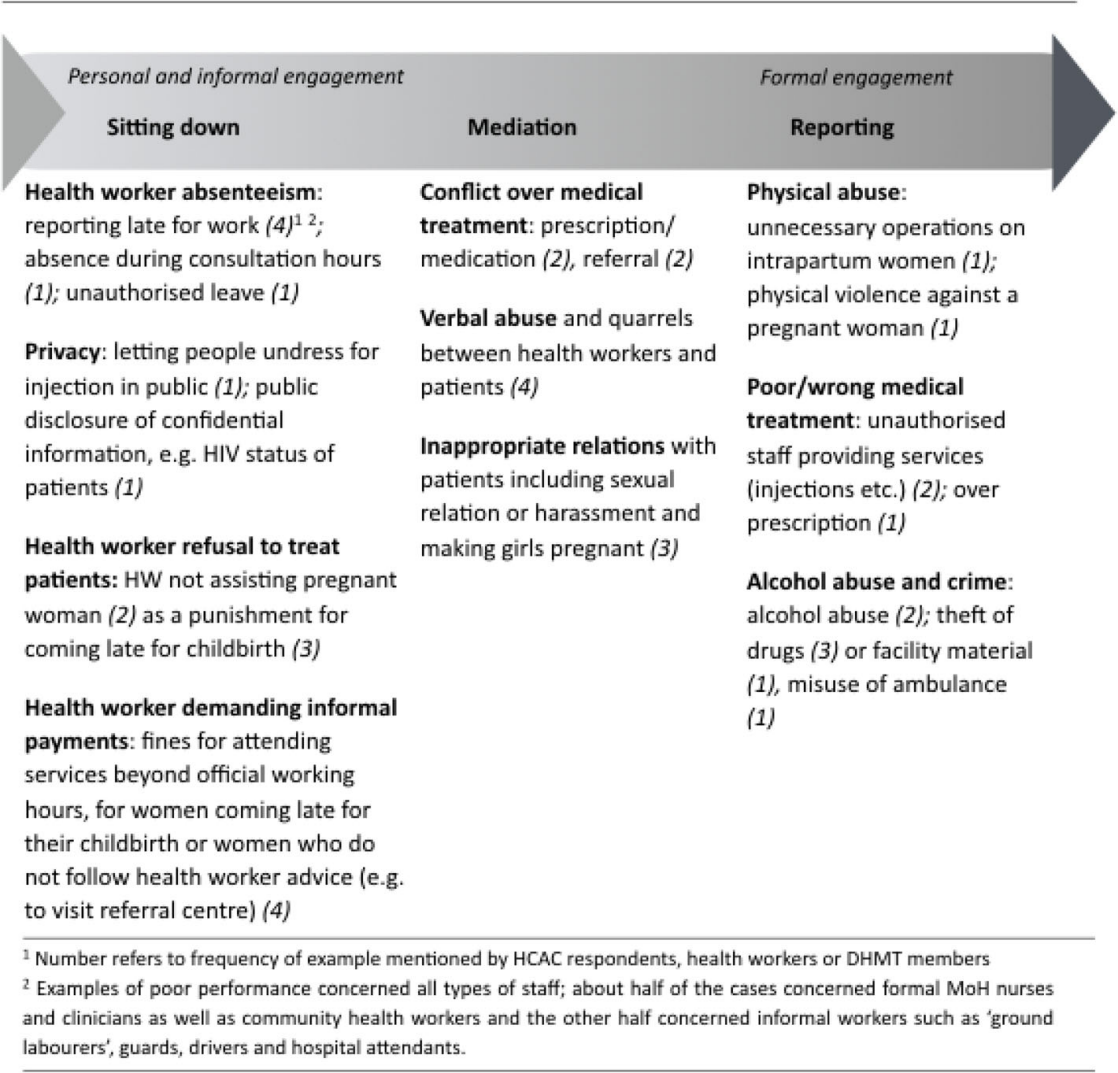
Approaches used by HCACs to address poor health worker performance

Both HCACs, health workers and district health authorities shared examples of cases of poor performance that were addressed by HCACs. From the analysis, two main approaches to responding to poor health worker performance emerged. First, HCACs address poor performance by engaging with health workers directly, through individual feedback (sitting down) and mediation. Secondly, they report poor behaviour to health authorities when the informal approaches do not lead to changed health worker behaviour or performance or when problems are considered beyond the authority of HCACs. Figure 1 presents each of these approaches with examples of the types of performance issues that HCACs address within each approach. In the following, the approaches and their outcomes are further described and analysed, illustrated by participants’ perceptions and quotes.

#### “Sitting down” and mediation

Fourteen out of 22 HCAC participants (63%) indicated that they meet health workers beyond formal meetings to discuss emergencies, problems or complaints. In the context of complaints, one HCAC member used the term “disciplining” to describe a case where he invited a health worker to “sit down and talk”. HCACs sit down with individual health workers or with both health workers and complainants. In the former they question, counsel, give feedback to and advice individual health workers; in the latter, they act as mediators and dialogue facilitators between health workers and patients and their families. Both strategies aim to share experiences, convey opinions and achieve a consensus. Notably, this strategy does not apply to all cases of observed or reported poor behaviour. Some HCACs first verify and investigate complaints, such as one HCAC that has established criteria for decisions on whether or not to use information: *“Our way is that here at the hospital the person who says ‘I heard …’, we don’t use that information but something that she has seen happening, we use”* (male HCAC treasurer).

A decision to sit down with a health worker is often taken by a small group of HCAC members, based on their judgement of the case. Individual feedback of HCAC members to health workers involves HCAC members – usually a chairperson or a delegation of two to three HCAC-members – approaching a health worker to meet individually soon after poor performance is observed or a complaint is received. One HCAC chairperson explained how members of the delegation are carefully selected as HCAC members *“differ in the way they keep secrets”.* This expression pertains to the view that not all members possess the right skills to confront health workers and ensure confidentiality of the conversation, a condition for “good” feedback and mediation that was mentioned by health workers too. In a feedback meeting, HCAC members present complaints or concerns and health workers are given the opportunity to provide their side of the story as one HCAC chairperson commented: *“we cannot know how they are thinking”,* and another: *“everyone is born with his or her own heart”*.

Mediation is used by HCACs in cases of overt disputes between patients and health workers at or around the health centre. HCAC members are called to intervene on the spot and act immediately. They then invite both parties *“to sit down and solve problems”*, and they use facilitation techniques to help participants *“view the problem from both sides”* and to *“teach both sides”.* A female HCAC chairperson, for example, regularly intervenes when patients are complaining about waiting times:

*“If the patients complain about long waiting hours…and they get angry, they call me. She [the nurse] works day and night so when the patients come we say sorry, but you should wait and also to the nurse that you should try to start working early morning when you can so that you can start helping the patients in good time”* (female HCAC chairperson).

HCACs use mediation to address health worker behaviour outside of the health centre, for example when a health worker discloses confidential information on patients in a public area or when (in all cases, male) health workers engage in sexual relations with women in the community. Sometimes the traditional chiefs are called upon to discipline health workers. In Malawi, chiefs play an important role in the protection and promotion of social and community norms and in dispute resolution, and they may address poor health workers’ behaviour towards community members.

Regardless of the type of mistreatment or abuse, most HCACs use non-confrontational tactics to discuss with health workers. For example, rather than admonishing health workers for late coming or absence, HCAC members provide advice for the future; health workers reported regularly receiving suggestions to start working early and always attend patients, also during the night. Similarly, in cases of verbal abuse or disputes, HCAC members advice health workers to receive patients in a friendly way. Only one HCAC member reported he had used a more confrontational approach to a health worker who provided injections to several people in one room, violating the privacy and dignity of the patients. He appealed to the law and accused the health worker of acting illegally.

#### Perceived changes as a result of HCACs feedback and mediation

HCAC and health worker participants observed mainly positive outcomes of HCACs efforts and often describe the outcomes in general terms: *“the problem was solved”,* or “*the health worker improved”, “the behaviour stopped”.* Results of sitting down include the agreement to improve or avoid similar behaviour, an explanation of behaviour (e.g. reasons for medical treatment, absence, refusal), or redress (acceptance of treating a patient who was previously denied treatment). Mediation has similar effects but also included apologies of either or both patient/family and health workers; avoidance of further quarrels; and settlement (for example financial compensation by the health worker for harm done to the patient or the community – including in the case of unaccepted sexual relations). In many cases, HCACs and health workers achieve consensus regarding the need to jointly mobilise resources for the health centre to facilitate respectful relations. Although outcomes such as answerability, receptivity, joint agreements and redress might not immediately translate into sustained quality improvements, they can be significant for individual service users and the functioning of the health centre.

HCAC members stated that only in a few exceptions, health workers deny or reject the complaint or advice or ignore mediation efforts. According to the following quote, without being specific, feedback and mediation can also cause health workers to stop working altogether:

*“Some people appreciate that we have done a good thing and others appreciate in secret, and others sometimes get angry. They wonder why we are telling them these issues; they say they don’t know anything, they just stay at home”* (male HCAC chairperson).

A HCAC member suggested that health workers’ responses depend on the staff position; in-charges, as health centre managers, are more likely to take comments than other staff such as young nurses or ground labourers, who do not accept a person at the same level (community) *“acting like a boss”.*

#### Reporting

When their initial approach of mediation, meeting and advice does not work out, or when *“the issue goes beyond”* the mandate of the HCAC, HCACs often reach out to local authorities, such as chiefs, the DHMT, or the local government council. In areas where the HCAC is less active, chiefs are often the ones reporting severe misbehaviour to other authorities.

The director of the DHMT in one of the districts explained that the DHMT receives up to two letters a week from HCACs, often co-signed by the health centre in-charge or the chief. Many letters are about a request to deploy more health workers, but some include demands to replace a misbehaving health worker. HCACs also contact the DHMT directly by phone, text messages or face-to-face. For example, a HCAC called the DHMT for immediate action against a nurse who was drinking at the bar during working hours, leaving patients unattended. In one case of alleged drug theft by a health worker, a HCAC chairperson called the DHMT and the police to ask for an immediate arrest. HCACs report mainly issues concerning professional health staff employed by the Ministry of Health and community health workers whereas offences committed by health centre support staff (ground labourers, guards, drivers, ward attendants) are dealt with by HCACs and chiefs themselves.

Responses by the DHMT vary from calling the health worker in question on the phone and asking for clarifications, to staging a formal investigation or addressing the complaint in a next supervision, as evidenced in HCAC meeting minutes and written responses by DHMT. The method and reaction speed depends, according to DHMT members, on the seriousness of the case and the resources available. At the time of the research, ten “serious” cases involving clinicians or nurses were handled by the DHMT. For six of them the outcome was pending; they were in the stage of official warning, investigation, temporary dismissal or rotation. Four health workers were transferred, of which two concerning cases of alcohol abuse, one of not reporting for work and one of the sale of health centre drugs.

### Perceived value of HCACs as social accountability interfaces

Study participants referred to individual feedback (sitting down) and mediation as the most appropriate way to address poor health worker performance for different reasons, related to the context in which each of the participants operates.

#### HCAC: value to “stay as children of one family”

Among HCAC participants, there was a general perception that, regardless of the type of poor performance, a dialogical approach in the private sphere would yield positive results. HCAC participants suggested that a health worker would “open up” if the HCAC person would *“dispose [him/herself] correctly to the health workers”,* and *“don’t impose fear”,* show that *“you are there for them, like a father to a son”*. Others stressed the need to *“approach health workers in a peaceful way”,* to *“come as a friend”* and to *“solve things peacefully in order to stay as children of one family”*. This alludes to the notion of “bridge” and “parent” reported previously and suggests HCACs are motivated to (re-)establish relationships rather than to control and rebuke individual health workers. A HCAC participant further indicated that the lack of evidence (for example on verbal abuse), due to the discretionary character of service provision, also required HCACs to use more constructive approaches to address (alleged) poor performance.

#### Health workers: personal and professional value of information and protection from public exposure

For almost all health workers, the HCAC is the preferred channel to receive information and complaints on performance, to *“know what is expected from me”* and *“what the community wants”,* as expressed by a nurse:

*“It [the HCAC] makes a difference because if you gave wrong services to a woman…, it makes me change how I work [..] because if they don’t tell me that this is not good, this is good, I cannot know.” (II nurse, female, HC17).*

One nurse positions HCACs as the only acceptable channel for feedback and mediation, in particular in the context of increasing use of social or traditional media that sometimes unfairly accuse health workers without proper analysis, harming their reputation, as well as that of other professionals. A male clinician felt that HCACs speak the truth, contrary to individual community members or service users who may *“just tell you something to please you”*, possibly referring to the power distance between health workers and service users through which service users are reluctant to voice their concerns directly. HCACs seem to be able to mediate the power distance by speaking both for communities and providers and channelling criticism in both directions. Health workers complain about being criticised in public and prefer HCACs to act as an intermediary to help avoid public exposure. According to a key informant, many health workers find being called to explain themselves to a community structure a shameful experience, and they take it seriously. Many health workers value the proximity of the HCAC (or at least some of its members) to the health centre as it provides the possibility for quick alerts and tailor-made responses to incidents. For many health workers, too, HCACs not only have a role in forwarding complaints but also in justifying health worker conduct or explaining the shortcomings (for example drug shortage and health worker instability) of the health centre to the community. In their view, such explanations could reduce expectations and prevent criticism, and shaming and blaming by the community.

#### District health authorities: value for the health system

DHMT members hardly have the time or resources to physically visit a health centre for supervision, let alone to resolve individual complaints or disputes. For DHMT members and poor interpersonal relations and attitudes in rural primary health centres are important to address, but out of their sphere of influence. For this reason, some DHMT members prefer HCACs solving disputes with health workers locally to reduce the DHMT workload and the burden on the under-resourced health system.

#### Relevance in the context of resource scarcity

DHMT members, health workers and HCAC members often referred to material and human resource shortage. They consider the lack of health workers and high workload as causes of health worker absence, verbal abuse or refusal to treat patients and the lack of equipment and housing as demotivating factors for health workers. Participants feel that health workers should not be held responsible or blamed for their behaviour since resource scarcity is related to constraints beyond the health centre level. In their efforts to address poor performance, participants expect HCACs to adopt a listening and supportive approach rather than a confrontational approach. HCAC actions to prospectively encourage good performance, prevent complaints from turning into conflict and providing personal care and support to health workers all fit the broader mandate of HFCs to support service provision and ensure a minimum level of acceptability of services.

### Perceived challenges of HCACs as social accountability interfaces

HCACs capacities to judge health worker performance, and the lack of clarity of roles and responsibilities in upward and downward reporting processes were mentioned as main challenges.

For about a third of health workers, HCACs were not as effective as they could be due to insufficient training and engagement with health centre staff. A health worker stressed that HCACs often cannot judge complaints and distinguish between personal frustrations and issues that affect the whole community. The main challenge concerned the criteria for reporting poor behaviour to health authorities. Participants reported a lack of clarity on reporting procedures and accountability relations between the HCAC, individual health workers, health centre management (officer in-charge) and the DHMT. Varying perceptions on criteria for reporting illustrate this. One HCAC chairperson stated that *“whenever a health worker does not understand us, we have to report”* and another suggested only cases of crime should be reported. DHMT members had different opinions: according to one DHMT member, the DHMT should be involved when issues are *“beyond the scope of the HCAC”,* while another one said that *“any problem with sub-standard patient care”* should be reported. The HCAC training manual on complaint management is not clear; it does not specify how, when and for what HCACs should forward complaints to the DHMT. This lack of clarity frustrates health workers as some feel HCACs sometimes report to the DHMT too quickly, without a proper investigation. At the same time, it leads to ambiguous reporting practices and to delays in addressing severe cases, for example when a HCAC waits with forwarding the issue or passing by other actors, such as chiefs, church leaders of local politicians. There is a paradox in that, on the one hand, DHMT appreciates the proxy-supervision role of HCACs, giving them the mandate and space to address localised issues, but on the other hand, DHMT and the government more generally, undermine the social accountability role of HCACs by providing little guidance and formal reporting instructions.

HCACs who do address poor performance through dialogue approaches at the local level feel they lack authority to follow through their initiatives; formally, health facility management is to inform the HCAC on conclusions and actions taken, but HCACs do not have clear guidelines on what to do if a health worker fails to do so. The same ambiguity applies to the responsibilities of HCAC to report to complainants or the community at large about how complaints were dealt with. From the data, it appeared that HCACs use their statutory meetings to share information among members, but it was not clear to what extent HCAC members informally or formally report back to their villages.

## Discussion

Despite the increasing amount of literature on social accountability in health systems, questions as to how citizens interact with health workers and demand accountability, remain largely unanswered, in particular with respect to the role of HFCs as facilitators of such interactions. This paper aims to add to the empirical evidence on the role of HFCs as social accountability interfaces by analysing the case of HCACs in rural Malawi. There are several common features in the functioning of the HCACs in the study.

First, the results reiterate the importance of intermediary structures, such as HFCs, to serve as vehicles for the identification and transmission of concerns from citizens and users to health worker, providers and authorities. The study shows that absenteeism and poor interpersonal relations, but also the denial of care and abuse, are recognised by HCACs as service delivery failures for which action is required. The awareness of entitlements and rights are preconditions for the expression of voice by individuals and groups of service users, and a minimum level of assertiveness is necessary to bring that voice forward [31]. On the other hand, few HCACs pro-actively collect information on performance, and few have systematised their monitoring function; their social accountability actions seem to rely on individual complaints or incidents rather than collective complaints. In addition, decisions to follow-up are often based on implicit judgments of a small group of HCAC members. They prioritise what happens to complaints and whether and how they approach health workers. The informal and personal nature of social accountability actions at the local level is consistent with the findings of other studies [2, 23]. HFCs, then, and probably particular members within HFCs, have substantial power to steer the accountability process and to determine the issues that are brought to the table and those that are not [32]. The ability of HFCs to raise and handle issues of quality of service delivery in an inclusive, collective and transparent way will determine whose voices are heard and whether health workers feel the social accountability actions are justified.

Second, the approaches HCACs use to call health workers to account can be qualified as ‘soft’ approaches that are based on personal relations, informal approaches to feedback, persuasion, negotiation and consensus building [33–35]. The experiences of HCACs illuminate the range of contextual factors that influence these approaches and point to the particular influence of resources. Participants perceived constructive approaches to dialogue as effective and relevant beyond the individual level. They see feedback processes as essential for the maintenance of social relationships around the health centre in a constrained health system. The scarcity of resources in the Malawian health system emphasises the need to develop capacities for collective problem solving rather than a control-based approach to improving the quality of services and health worker performance. According to a classification of social accountability purposes by Wetterberg, Brinkerhoff, & Hertz (2016), the purpose of HFC’s social accountability role can be understood as “co-production focused”. This purpose moves away from compliance-focused or confrontational actions to interrogate and sanction health worker behaviour [36]. It also deviates from the retrospective character of many accountability approaches; HFCs combine multiple service delivery functions and build relations with health workers through other activities than only monitoring and feedback on poor performance. Hence, while the initial framework of this study emphasised the retrospective approach to social accountability, the results reiterate the value of a prospective and interactive approach to social accountability at the local level and the fact that “being watched” may be a sufficient incentive for health workers to perform, as suggested by accountability scholars [37].

At the same time, constructive dialogue provides no guarantee that health workers take a positive stance towards HFC involvement in service monitoring; they can as well deny certain behaviours, neglect advice and not take responsibility as suggested in our study and elsewhere [18]. Also, ‘soft’ approaches alone are often unable to deal with the lack of responsiveness of providers, serious cases of misbehaviour, illegal or inappropriate actions and transgressions in behaviour [38] for which an authoritarian mode of accountability is required, operating simultaneously with the co-production mode of accountability [39].

Third, and following the previous point, the study observed a lack of clear upward reporting procedures and the lack of DHMT responsiveness to the issues raised by HCACs. Although DHMTs see a value in HCACs performing proxy-supervision, their lack of guidance and engagement with HCACs prevents HCACs from extending their social accountability role and power beyond dealing with localised issues of health worker performance. This is a common challenge in social accountability efforts: the vertical integration of social accountability that enables complaints at the local level to be systematically and effectively addressed ‘upwards’ in the health system [40].

### Implications for policy and practice

The strengthening of HFCs social accountability role at the local level and the optimisation of vertical integration requires actions at multiple levels. First, it needs a distinguishable mandate of citizens groups (in this case HFCs) in the accountability landscape, including the types of issues HFCs could monitor. In Malawi, the ‘Charter of Patients’ and Health Workers’ Rights and Responsibilities’ and the ‘Charter on Safe and Respectful Maternity Care’ [41, 42] have been widely disseminated, also among HCACs, and they could be used as starting points for social accountability. Second, investments in the quality and principles of accountability processes are needed. This involves the strengthening of capacities of both HFCs and health workers to conduct broad-based community consultation on perceived and experienced care and to strengthen dialogue and negotiation skills, documentation and transparent reporting procedures. The role of statutory HFC meetings as central forums of accountability and spaces of negotiation could be enhanced. HFC capacity strengthening strategies should take a holistic perspective; the findings support observations in earlier research that HFCs are heterogeneous entities with multiple roles, responsibilities and functions and that they are confronted with diverging expectations from communities, service users, health workers, and health authorities [1, 2]. The accountability role of HFCs should be understood in this context; it is part of a more comprehensive set of activities HFCs perform to support local health service delivery which varies per context. A focus on strengthening HFCs capacities in monitoring or complaint management would be a too narrow approach.

Third, in order to enhance vertical integration, reporting and responsiveness mechanisms need to be clarified between HFCs and district authorities. Furthermore, the linkages between social accountability and service delivery programming, supervision and evaluation and quality improvement programmes can be improved. For example, the role of community structures such as HFCs in the provision and monitoring of services could figure more prominently in national sexual and reproductive health policies or quality improvement strategies. This would be more effective than strengthening the accountability interface role of HFCs as a stand-alone project. The strengthening of social accountability relations requires long-term repeated and extended interactions between citizens, health workers and provider organisations [36].

Finally, there is no doubt that continued investment in material and human resources for health services will be essential for both the performance of health workers and the effectiveness of social accountability.

### Strengths and limitations

Despite the careful introduction of the topic, some participants might have felt uncomfortable talking about challenges in health worker performance. This is complicated by the fact that few HCACs have documented evidence of their practices, which hampered our understanding of their activities over time. We also did not collect the opinion of the service users involved in the cases, and hence we do not know how they evaluate the representativeness, accessibility, and effectiveness of HCACs as social accountability interfaces. We have an unclear picture of the frequency with which service users express their concerns or how often HCACs observe and address poor performance; whereas some HCACs stated they do so every day, others indicated they rarely do, and some participants shared scenarios rather than experienced events. An analysis of social hierarchies, including gender relations, could have contributed to a more nuanced view on who expresses concerns (and who does not) and whose concerns and interests are addressed. Discussions with key informants and DHMT members, however, confirmed most of the findings. For the analysis of the examples in Fig. 1, we used data source triangulation to strengthen reliability; both HCAC and health worker participants needed to have shared the same example for inclusion as a case. Data from HCAC meeting minutes and interviews with DHMT members, in particular under the category of ‘reporting’ confirmed some cases. Therefore, we estimate that the content of complaints and performance issues as presented in Fig. 1 represent a reliable picture of the types of problems that service users are confronted with at a health facility. A feedback meeting with the DHMT at the end of the data collection phase helped to check the credibility of our preliminary interpretation and conclusions.

The sample of 22 HCACs was deemed sufficient to gain a comprehensive overview of perceptions on, and experiences with HCAC as social accountability interfaces. No new insights were gathered after interviewing the key persons around each HCAC (chairperson and 1–2 health workers). Our findings likely represent the situation of other HCACs in the district and to some extent in the Northern Region. Key informants, who had experience with HCACs in other regions in Malawi, held that HCAC members’ in Northern Malawi had relatively high levels of literacy, education and knowledge of the health sector, explaining their active engagement with health workers. This was associated with the overall education levels in the Northern region but also with regular training and support from district health authorities and NGOs.

## Conclusions

The study explored the approaches HFCs use to call health workers to account on their behaviour and performance, and it reviewed the social and organisational context in which HFCs operate. The findings add to the knowledge base of social accountability and HFC studies. The primary function of social accountability efforts by HFCs in this study is the management of social relations around the health centre through an informal, constructive approach rather than correcting individual health workers. This function is essential for the establishment of people-centred health systems and efforts to achieve universal health coverage. The potential and limitations of HFCs in improving health worker performance should be assessed from a broader perspective and in comparison to other efforts to improve the quality of services and address poor performance and illegal actions by health workers.

## Additional file

Additional file 1: Features of the HCACs in the study. (DOCX 14 kb)

## Abbreviations

CHAM: Christian Health Association of Malawi
DHMT: District Health Management Team
HCAC: Health Centre Advisory Committee
HFC: Health Facility Committee
