# Supplementary material for: “We come as friends”: approaches to social accountability by health committees in Northern Malawi
Source: BMC Health Serv Res. 2019 May 2;19:279. doi: 10.1186/s12913-019-4069-2 (PMC6498677; doi:10.1186/s12913-019-4069-2)
Supplement: Supplementary file 1 — Features of the HCACs in the study. (DOCX 14 kb) [file 12913_2019_4069_MOESM1_ESM.docx]

**Additional file 1.** Features of the HCACs in the study

|  | | Total |
| --- | --- | --- |
| Total HCAC’s included in study | | 22 |
| Composition | |  |
| Members | 10  <10  >10 | 18 (82 %)  3 (13.5%)  1 (4.5%) |
| Chairperson | Male  Female | 21 (95.5%)  1 (4.5%) |
| Vice- chairperson | Male  Female | 15 (68.2%)  7 (31.8%) |
| Women equally represented in HCAC | Yes  No, men > women No, women > men | 4 (18.2%)  15 (68.2%)  3 (13.6%) |
| Secretary being health care provider | Yes, the in charge of the facility  Yes, other staff  No | 8 (36.3%)  3 (13.7%)  11 (50%) |
| Health care provider usually present in meetings | Yes  In half of the meetings  When they are called  Never | 13 (59.1%)  3 (13.6%)  4 (18.2%)  2 (9.1%) |
| Statutory and informal meetings | |  |
| Scheduled formal meetings | Twice a week  Once a month  Twice a month  Once every 3 months | 1 (4.5%)  9 (40.9%)  10 (45.5%)  2 (9.1%) |
| HCAC meets health care providers besides statutory meetings | Yes, in case of emergency/problem  Yes, when HCAC visits health center  Yes, when HCAC calls providers  No, never  Unknown | 14 (63.6%)  3 (13.7%)  1 (4.5%)  1 (4.5%)  3 (13.7%) |
| Presence/visibility in the health centre | | |
| HCAC physically present in the health center | Daily  2/3 times a week  Once a week  Once a month  Never  Unknown | 5 (22.7%)  6 (27.3%)  2 (9.1%)  5 (22.7%)  3 (13.7%)  1 (4.5%) |
| Support |  |  |
| HCAC received training | Yes  No | 16 (72.7%)  6 (27.3%) |
| HCAC received allowance | Yes  No | 0 (0%)  22 (100%) |

Data are n (%)
